# Regional and Temporal Variation in Receipt of Gabapentinoid and SSRI/SNRI Therapy Among Older Cancer Survivors in the United States

**DOI:** 10.3390/curroncol32100576

**Published:** 2025-10-17

**Authors:** Amber Nguyen, Yong-Fang Kuo, Daoqi Gao, Mukaila Raji

**Affiliations:** 1 School of Medicine, University of Texas Medical Branch, Galveston, TX 77555, USA; 2 Department of Biostatistics & Data Science, University of Texas Medical Branch, Galveston, TX 77555, USA; dagao@utmb.edu; 3 Geriatric Medicine Division, Department of Internal Medicine, University of Texas Medical Branch, Galveston, TX 77555, USA; muraji@utmb.edu

**Keywords:** gabapentin, cancer survivor, SSRI, SNRI, pain management

## Abstract

Opioids and benzodiazepines (BZD) are commonly prescribed for older cancer survivors with co-occurring pain and anxiety. The prescribing rate of gabapentinoids (GABA), Selective Serotonin Reuptake Inhibitors (SSRIs), and Serotonin-Norepinephrine Reuptake Inhibitors (SNRIs) in the general population has increased as opioid/BZD alternatives, but little is known on temporal/regional trends in use of these alternatives among older cancer survivors. A retrospective cohort study using SEER-Medicare data was conducted. Patients aged ≥ 66 years, diagnosed with breast, colorectal, prostate, or lung cancer as their first cancer diagnosis any time from 2000 to 2015 and who were alive more than 5 years after cancer diagnosis, were eligible for inclusion. Temporal trends varied by region (*p* < 0.0001) and opioid-naïve status (*p* < 0.0001). Compared to 2013, GABA and SNRI use increased, while BZD and opioid use decreased. All regions experienced declines in opioid use. From 2013 to 2018, all regions saw an increase in GABA use, with a decline in 2020. GABA prescriptions increased more in opioid-naïve groups compared to non-opioid-naïve patients. The yearly trends in GABA and SSRI/SNRI use varied by region among older cancer survivors. Clinical practice variation suggests needs for further research on improving consistency and quality of cancer care.

## Simple Summary

Many older cancer survivors experience pain and anxiety, which are often treated with opioids and benzodiazepines. However, due to concerns about the risk of these medications, doctors have increasingly prescribed alternatives such as gabapentinoids (e.g., gabapentin) and antidepressants such as SSRIs and SNRIs. While these medications are becoming more common, little is known about how their use has changed over time and whether prescribing patterns vary across different regions in the USA. We analyzed data from SEER-Medicare on older patients diagnosed with breast, colorectal, lung, or prostate cancer who had survived at least five years after diagnosis. Our results showed that the use of gabapentinoids and SNRIs increased over time, while opioid and benzodiazepine use declined. These findings highlight important shifts in medication use among older cancer survivors. Differences between regions suggest that prescribing practices vary across the country, which may reflect differences in medical training, policies, or care accessibility.

## 1. Introduction

Chronic pain and anxiety are common co-occurring conditions among cancer survivors aged ≥65, a population that constitutes 67% of all cancer survivors in the US [1]. Compared with the US general population, cancer survivors have approximately double in prevalence of chronic and high-impact pain and experience higher rates of anxiety and depression. Current ASCO guideline recommends routine screening and evidence-based management of these symptoms [2,3]. Chronic pain in cancer survivors can last for years, even after completion of cancer treatment, with prevalence, type, and severity of pain varying by patient demographics, cancer types, prior cancer treatments, and types of pain treatment (including opioids and non-opioid therapy) [4,5,6,7,8,9,10,11,12]. Prescription opioids used during cancer treatments are frequently continued even after the completion of cancer treatment [4]. As such, opioid usage rates among the cancer survivor population are higher than those of the non-cancer population [13]. While short-term opioids are key to multimodal pain treatments for cancer pain management, long-term opioid therapy (≥90-day supply of opioids/year) in older adults is associated with serious adverse drug events: falls, delirium, hypogonadism, infections, and cardiorespiratory complications, among others [14,15,16,17].

Awareness of opioid-related toxicities by patients and prescribers and various opioid-related policies have led to a decline in long-term opioid use among older cancer survivors [17]. In the general non-cancer population, research shows a shift toward increased use of non-opioid analgesics (e.g., gabapentinoids [GABA]), whose prescribing increased in every state from 2009 to 2016. State-specific prevalence rates in 2016 ranged from 12.7 to 43.9 per 1000 beneficiaries, with higher prescribing rates observed in the South [18]. Due to the efficacy of GABA and Serotonin-Norepinephrine Reuptake Inhibitors (SNRIs) in treating the neuropathic and chronic widespread pain, these medications have become increasingly relevant in the treatment of adult cancer survivors over other non-opioid and benzodiazepine (BZD) alternatives, such as non-steroidal anti-inflammatory drugs (NSAIDs) and acetaminophen [16]. Contemporary guidelines emphasize non-pharmacologic strategies (e.g., exercise, physical therapy, cognitive-behavioral approaches), as first-line for many chronic pain conditions with non-opioid medications considered when indicated and opioids reserved for select circumstances [16]. While non-opioid pharmacologic options are often considered in survivorship, GABAs and SNRIs carry their own age-salient risks in older adults. Gabapentinoid-associated dizziness, sedation, gait imbalance, especially with renal impairment or co-prescribed with CNS suppressants, and SNRI-associated hyponatremia and falls are risks that necessitate individualized assessment [19,20].

Likewise, co-occurring anxiety in cancer survivors with chronic pain is frequently treated with BZD (with or without opioids), a prescribing practice linked to a high rate of falls/fracture and opioid overdose [4,21]. The high risks of toxicities of such opioid/BZD prescribing prompted the 2016 Centers for Disease Control and Prevention (CDC) guideline recommendation of using non-opioid and non-BZD alternatives as first line therapy for pain and anxiety, and avoiding opioid/BZD co-prescribing. Additional policy changes at state, health system, and health payer levels have been accompanied by an increase in prescribing of non-BZD medications (e.g., Selective Serotonin Reuptake Inhibitor [SSRIs]/SNRIs) in the general population, but data is limited in the older cancer population [21]. Significant regional variation in the use of SSRIs and SNRIs exist across the US, with use ranging from less than 1% to more than 40% of residents in different postal code areas [17,22]. While data exist on geographical and temporal trends in prescribing of non-opioid analgesics (GABA) and non-BZD anxiolytics (SSRI/SNRIs) in the general population, little exist on temporal and regional trends in receipt of GABA and SSRI/SNRIs in older cancer survivors.

We thus examined the annual rates (from 2013 to 2020) in the receipt of long-term GABA and SSRI/SNRI for older persons with a history of breast, colorectal, lung, or prostate cancer diagnosis. We also examined whether the temporal trends varied by opioid naïvety, given that patterns of opioid use are influenced by previous opioid use among older cancer survivors [4]. It is unclear whether the trend towards prescribing of non-opioid analgesics and non-BZD anxiolytics in the non-cancer population extends to older cancer survivors, or whether this trend has been consistent across regions. We explored the extent to which state and federal policies affected prescribing practices among older cancer survivors, evaluating changes year-by-year and by region. Local factors may be driving prescriber responses to these policy changes. As older adults are more vulnerable to medication-related harms (due to age-related decline in drug metabolism and high rate of multiple chronic conditions), we focus on older cancer survivors, as they often face persistent neuropathic pain, unique mental and physical health challenges, and a high rate of geriatric syndromes (e.g., falls, osteoporosis, frailty), underscoring the need to understand factors associated with prescribing of safer, effective and age-friendly medications in this population [23]. With the high rates of cancer- and treatment-related neuropathic chronic pain syndrome and anxiety in older cancer survivors, it is important to examine practice-and-policy actionable factors associated with prescribing of non-opioid and non-BZD treatments (e.g., GABA and SSRIs/SNRIs). Examining regional and temporal variations in GABA and SSRI/SNRI therapy can improve our understanding of barriers and facilitators to prescribing of the safer non-opioid/non-BZD drugs in older cancer survivors, a population at high risk of adverse drug events due to age-related decline in drug metabolism, high rates of multiple chronic conditions (multi-morbidity), and polypharmacy.

## 2. Materials and Methods

### 2.1. Data Source

A retrospective cohort study was conducted using linked Surveillance, Epidemiology, and End Results (SEER)—Medicare datasets [24]. The dataset contains population-based cancer registries from 22 SEER regions, covering approximately 48.0% of the US population. The claim data includes Parts A and B, which cover inpatient hospital stays and outpatient services, respectively. Medicare Part D provides coverage for outpatient prescription drugs. The institutional review board of the University of Texas Medical Branch (IRB: #24-0105) approved this study on 29 March 2024. This study complies with the RECORD guidelines [25].

### 2.2. Study Cohort

Patients diagnosed with breast, colorectal, prostate, or lung cancer as their first cancer diagnosis any time between 1 January 2000, and 31 December 2015 were eligible for inclusion. Persons were excluded from the study if they were diagnosed at autopsy or on a death certificate. To select cancer survivors, we limited our study cohort to patients without a second primary diagnosed cancer, and alive more than 5 years after cancer diagnosis, characteristics which were most consistent with the definition of a long-term survivor, proposed by Surbone [26].

Annual cohorts were constructed for each year of the study (2013–2020) using consistent inclusion and exclusion criteria. For each annual cohort, individuals younger than 66 years on January 1 of the corresponding year were excluded. To ensure complete information, we included individuals who had continuous Part A, B, and D enrollment and no enrollment in a Health Maintenance Organization (HMO) in the 12 months prior to January 1 of the corresponding year, and during the 12 months of follow-up or until death. Patients who died during the follow-up period are censored in the analysis. Additionally, those who received cancer treatment (chemotherapy and radiotherapy) in the 12 months prior to January 1 of the corresponding year or who died in January of the corresponding year were excluded. Since we defined 30-day drug prescription as using the medications, patients who died in January were excluded for complete data purposes. Excluding prior treatment (chemo- or radio-therapy) in the 12 months prior ensured that medication used during the study period was not driven by active or recent treatment. This approach allowed us to focus on stable prescribing patterns in the survivorship phase, rather than acute prescribing related to active cancer management or peri-treatment recovery. Cancer treatments were defined using International Classification of Diseases (ICD) codes from Medicare Provider Analysis and Review, outpatient, and carrier files and Current Procedural Terminology/Healthcare Common Procedure Coding System codes from outpatient, Durable Medical Equipment and Carrier files.

### 2.3. Outcome Variables

National Drug Codes from RedBook were used to identify GABA, BZD, SSRI, SNRI, and opioid prescriptions from the Part D Event file. The cumulative number of calendar days a person possessed a drug prescription in a calendar year was calculated individually. Any patient who had at least one 30-day prescription for a type of studied medication was categorized as using this medication. Patients who had multiple back-to-back or overlapping prescriptions of one type of medication, with >30 cumulative days of medication use without a >30 days gap between two medications, were also categorized as using this medication.

### 2.4. Covariates

Time-invariant covariates were gender, race and ethnicity, diagnosis cohort, cancer diagnosis, and original reason for Medicare entitlement. Diagnosis cohort was the recategorization of the year of cancer diagnosis into 4 cohorts: 2000–2003, 2004–2007, 2008–2011, and 2012–2015.

Time-varying covariates were the years post-cancer diagnosis, age at the beginning of a calendar year, metropolis status, census region (West, Northeast, Midwest, South), dual eligibility, Charlson’s comorbidity index score > 1, depressive disorder, anxiety disorder, alcohol use disorder, drug use disorder, and opioid-naïve status. Opioid-naïve cancer survivors were individuals who received no opioid prescriptions in the 12 months prior to January 1 of a calendar year. Mental health disorders were identified based on appropriate ICD-9 and ICD-10 diagnostic codes using the Chronic Conditions Warehouse algorithm.

### 2.5. Statistical Analysis

Means (standard deviations [SD]) and medians (interquartile range) were calculated for continuous variables, and frequencies (percentages) were calculated for categorical variables. Descriptive analysis was conducted to examine the distribution of patient characteristics by calendar year to assess how the composition of the overall cohort changed over time. In order to assess crude drug use differences across years, we calculated prevalence rates for each calendar year by dividing the total number of persons who received the drug by the total number of person-years contributed for each calendar year. For each calendar year, a person could contribute from a minimum of 1/12 person-years to a maximum of 1.0 person-years.

Multivariable analysis estimating the adjusted odds ratio (aOR) of receipt of drug within each calendar year was conducted utilizing generalized estimating equations (GEE) with a binomial distribution, logit link function, and an autoregressive (AR1) correlation structure to account for repeated measures of persons. Since the repeated measures have evenly spaced time intervals, the AR1 structure is appropriate to use. An offset statement was included for the person-years contributed to each calendar year. This GEE model was chosen because the study was not only interested in the trends, but also in the characteristics of the study populations. To assess whether time trends differed across US regions and opioid naïve status, we tested statistical interactions between the calendar year and census region, and the calendar year and opioid naïve by including each individual interaction term into the main-effects model. Since both interactions were significant for most drug outcomes, models were stratified by these variables to examine how temporal trends in receipt of drugs varied by regions and opioid naïve status. Prevalence rates for each drug, stratified by regions across years, were also calculated. All statistical analyses were performed with SAS version 9.4 (SAS Institute, Inc., Cary, NC, USA).

## 3. Results

Overall, the study included 704,766 persons who contributed a total of 2,854,504 person-years. The minimum number of person-years contributed by a single individual was 0.167 and the maximum was 8.0, with an average of 4.05 person-years (SD = 2.29) and median of 4.00 person-years (Q1, Q3 = 2.00, 6.00).

Table 1 demonstrates how the person characteristics changed at selected years during the study period. The mean age of the sample increased from 77.6 years in 2013 to 78.2 years in 2020, with the median age remaining consistent at 77.0 years throughout the study period. The number of years post-cancer diagnosis increased, with the mean rising from 7.6 years in 2013 to 12.0 years in 2020. Males comprised around 53% and females around 47% of the sample each year.

Racial/ethnic composition showed a slight decline in the percentage of Hispanic and non-Hispanic Black (NHB) participants. Cancer diagnosis distribution saw a decrease in colorectal cancer survivors from 17.6% in 2013 to 14.5% in 2020, while prostate cancer survivors consistently comprised around 44–45% of the sample. From 2013 to 2020, the geographic distribution of participants remained stable in the Northeast, with a slight decrease in the Midwest and South regions, and a slight increase in the West region. Urban–rural status revealed an increase in metro residents from 85.0% in 2013 to 87.2% in 2020. The percentage of participants with depressive and anxiety disorders increased over the years. The percentage of opioid-naive participants rose from 67.3% in 2013 to 77.2% in 2020.

**Figure 1:**
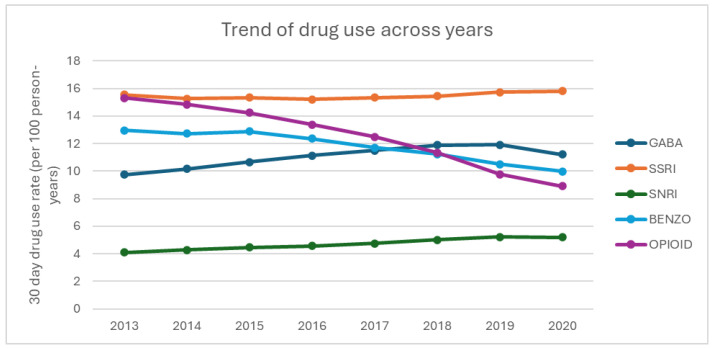
Trends in medication use among older cancer survivors (2013–2020).

Figure 1 displays the rate of medication use with at least 30-day prescription, stratified by drug. BZD and opioid medication use decreased, while GABA and SNRI medication use increased. SSRI use remained stable, with minor fluctuations.

Table 2 shows calendar year trends of increasing likelihood of GABA and SNRI prescriptions, peaking in 2019 (aOR for GABA: 1.40, 95% Confidence Interval [CI]: 1.35–1.45; aOR for SNRI: 1.25, 95% CI: 1.18–1.32), while the odds for BZD and opioid prescriptions decreased over time, particularly for opioids in 2020 (aOR: 0.59, 95% CI: 0.57–0.62). Age was a notable factor. Survivors aged 75–84 had increased odds of receiving GABA, SSRI, and opioid medications, while those aged 85+ were less likely to receive BZD and SNRI but more likely to receive SSRI and opioid medications. Females had higher odds than males of being prescribed all medication types, particularly SSRI (aOR: 1.73, 95% CI: 1.68–1.77) and SNRI (aOR: 1.86, 95% CI: 1.77–1.95).

Racial and ethnic disparities were evident. NHBs had lower odds of receiving BZD, SSRI, and SNRI but similar odds of GABA and opioid medications as Non-Hispanic Whites (NHWs). Hispanics also had lower odds than NHWs for BZD, SSRI, SNRI, and opioid medications. Cancer diagnosis type influenced medication receipt; prostate cancer survivors had lower odds of all medication types compared to other cancer types, while breast cancer survivors had higher odds for SNRI (aOR: 1.11, 95% CI: 1.06–1.15).

Geographic region had varied effects; survivors in the South had higher odds for all medications except Opioids, while those in the Northeast had lower odds for GABA and higher for BZD. Non-metro residents had higher odds for GABA and opioid medications, but lower odds for BZD and SNRI. Survivors who originally qualified for Medicare via disability had higher odds for all medication types, and dual eligibility status was associated with increased odds for all medications, particularly opioids (aOR: 1.70, 95% CI: 1.68–1.73). Charlson comorbidity index score ≥ 1 was linked to higher odds of receipt for all medications. Mental health conditions were strong predictors of medication use; survivors with depressive disorder had higher odds of SSRI and SNRI use, and those with anxiety disorder had higher odds for BZD use. Alcohol and drug use disorders also influenced medication receipt; drug use disorder increased the odds for opioid prescriptions (aOR: 2.79, 95% CI: 2.73–2.86). Opioid-naive cancer survivors had lower odds of receiving opioids (aOR: 0.14, 95% CI: 0.14–0.14).

**Figure 2:**
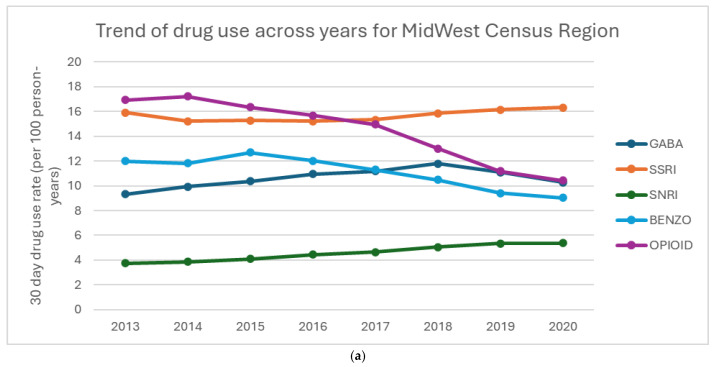

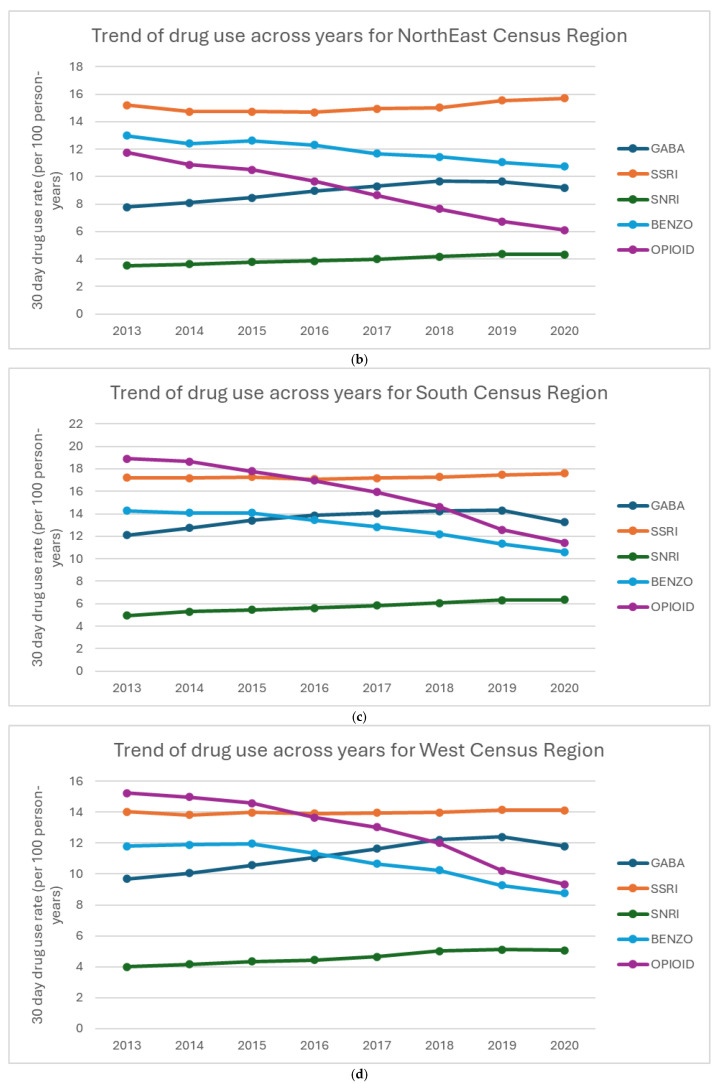
(**a**–**d**) Percentage of drug use by region: (**a**) Midwest Census Region, (**b**) NorthEast Census Region, (**c**) South Census Region, (**d**) West Census Region.

Figure 2a–d show the percentage of drug use for each region. Overall, BZD use decreases over time in all regions. All regions also saw notable declines in opioid use, with the Northeast showing the most significant reduction. From 2013 to 2018, all regions had a steady increase in GABA use, with a decline in use in 2020. SSRI use was fairly stable across all regions, with a slight increase in both SSRI and SNRI use. The South shows a higher prescribing pattern across all the drugs compared to West, Northeast, and Midwest regions.

**Figure 3:**
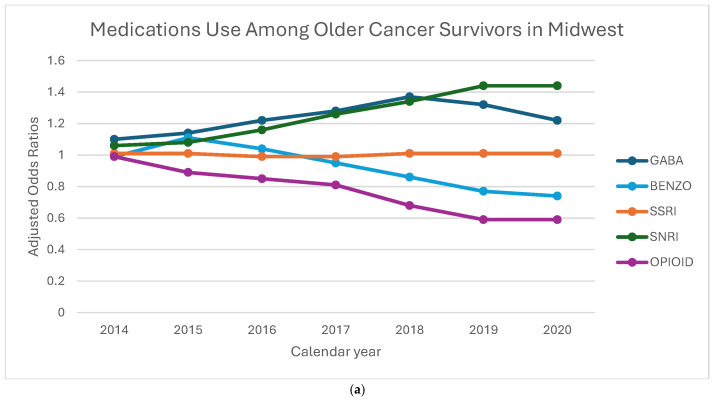

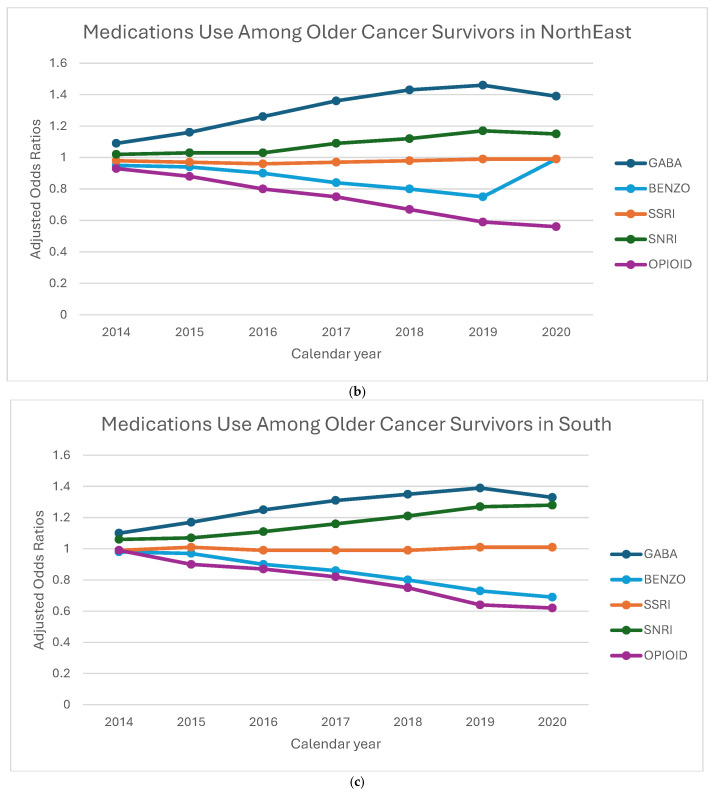

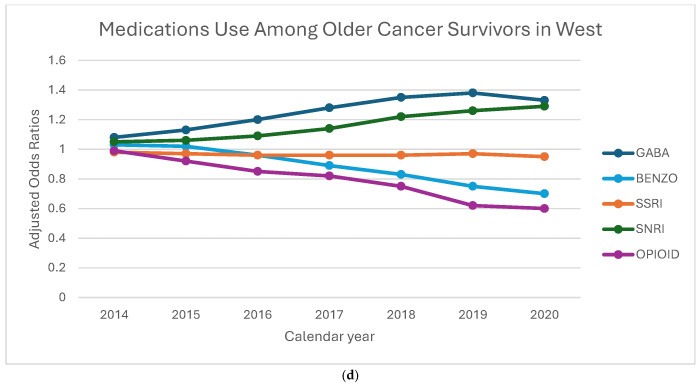
(**a**–**d**) Adjusted odds ratios for medication receipt by region and year, 2013–2020. To preserve legibility, CIs are de-emphasized in the figure; full 95% Cis and linear trend estimates (calendar year modeled as a continuous term, with region-year interaction) to Addendum Table 1.

Figure 3a–d displays the aORs of receiving of GABA, BZD, SSRI, SNRI, and opioid medications among older cancer survivors, stratified by geographic region and calendar year. GABA prescriptions steadily increased across all regions, with the Northeast showing the highest odds in 2019 (aOR: 1.46, 95% CI: 1.36–1.56). SNRI prescriptions also showed a consistent rise, with the largest increase in the Midwest in 2020 (aOR: 1.44, 95% CI: 1.12–1.86), while the Northeast saw a more modest rise compared to other regions. BZD prescriptions declined over time, but the decline was less pronounced in the Northeast and Midwest compared to the West and South. Opioid prescriptions saw the steepest reductions in the Northeast and Midwest, with the lowest odds in 2020. SSRI prescriptions remained relatively stable across all regions, showing minimal variation. These patterns suggest a shift toward increased use of GABA and SNRI medications, with regional differences in the decline of BZD and opioid prescriptions, highlighting the influence of local prescribing practices and policies. Linear trend estimates and full confidence intervals are provided in Supplementary Table S1.

Table 3 highlights the prescription trends among opioid-naïve and non-opioid-naïve older cancer survivors. For opioid-naïve patients, GABA prescriptions increased more markedly, peaking in 2019 (aOR: 1.62, 95% CI: 1.54–1.69), compared to non-opioid-naïve patients (aOR: 1.32, 95% CI: 1.25–1.38). Opioid-naïve patients also experienced a sharper decline in opioid prescriptions, with odds falling to 0.47 (95% CI: 0.45–0.50) in 2020, whereas in the non-opioid-naïve group, the odds decreased more modestly to 0.72 (95% CI: 0.69–0.76).

In contrast, BDZ use decreased more significantly in non-opioid-naïve patients, with the odds dropping to 0.61 (95% CI: 0.57–0.65) by 2020, compared to opioid-naïve patients (aOR: 0.76, 95% CI: 0.72–0.80). Additionally, while SSRI use decreased more among non-opioid-naïve patients, showing slightly lower odds over the years, it remained relatively stable in opioid-naïve patients. Lastly, the rise in SNRI prescriptions was comparable between groups, showing a steady increase by 2020 in both opioid-naïve (aOR: 1.27, 95% CI: 1.17–1.38) and non-opioid-naïve patients (aOR: 1.30, 95% CI: 1.19–1.41).

## 4. Discussion

We observed substantial regional and temporal differences from 2013 to 2020 in the prescribing of different analgesic and anxiolytic medications for co-occurring pain and anxiety disorders among long-term cancer survivors. Overall, we found a rise in GABA and SNRI prescribing and a decline in BZD and opioid prescribing. These changes likely reflect multiple factors, including the impact of the 2014 Drug Enforcement Administration hydrocodone rescheduling, the 2016 CDC opioid prescribing guidelines, and various regulations at state, healthcare payer, and health system levels. The relative stability in SSRI prescribing over time was unexpected, as we anticipated that SSRIs use for anxiety would rise as BZD use declined, due to the better safety profile of SSRI. Our findings of stable SSRI use over time in older cancer survivors are different from prior findings that showed an upward trend in SSRI usage in the general population, from 2000 to 2020, especially at the onset of the COVID-19 pandemic [27,28].

Past studies showed that regional practices, policy changes at state and federal levels, and prescribing behaviors affect prescribing trends of central nervous system (CNS)-acting medications [21,29,30,31]. The increasing trend in GABA and SNRI use, and the declining trends in BZD and opioid use, suggest a shift in prescribing preferences or practices over the years. The decline in opioid use likely reflects myriad policies/laws aimed at curbing opioid overprescribing, a major contributor to opioid misuse/addiction and overdose [32]. Similarly, the decline in BZD use may be attributed to various medical society practice guidelines/government policies and the increasing awareness about the risks (e.g., falls, cognitive decline) associated with long-term BZD use. Reinforcing these trends through federal guidelines (e.g., CMS guidelines) provides an opportunity to promote consistent prescribing practices across regions.

Conversely, the increased use of GABA and SNRI medications may reflect a shift toward these drugs as presumably safer alternatives for managing pain and anxiety. In particular, the rise in SNRI prescriptions likely reflects its dual roles in treatment of both pain and anxiety, thus reducing polypharmacy. This is particularly relevant to older cancer survivors as SNRIs—potent anxiolytic agents—are also FDA-approved for treatment of neuropathic pain (a common issue in cancer survivors) and musculoskeletal pain syndromes [32,33,34,35]. While SNRIs are guideline-supported options for neuropathic pain, their analgesia effect is modest in trials, and many older survivors continue to experience pain-related burden; thus, use may reflect a preference for a comparatively safer risk profile [36,37]. Perceived safety is likely to contribute to uptake. Nevertheless, GABA and SNRIs are not benign in older adults. GABAs can cause dizziness, sedation, and falls, while SNRIs raise concern for hyponatremia and falls [19,20].

The slight reduction in GABA prescriptions across regions in 2020 in older cancer population is unexpected, given prior research showing an increase in GABA use in the general population during the COVID-19 pandemic across many clinical settings [38,39]. This decline can potentially be due to a shift toward non-pharmacological approaches for managing chronic cancer pain, such as physical therapy, acupuncture, or injections; this is an area for future study. Additionally, policy changes like the reclassification of GABA to Schedule V in states such as Kentucky, West Virginia, and Tennessee, along with its inclusion in prescription drug monitoring programs in several states like Kansas, Massachusetts, Minnesota, Nebraska, New Jersey, North Dakota, Ohio, Virginia, and Wyoming from 2013 to 2018, may have contributed to reduced GABA use [30]. Furthermore, the 2016 American Society of Clinical Oncology guideline recommended NSAIDs and acetaminophen as effective non-opioid alternatives for cancer pain management, with NSAIDs (e.g., diclofenac and ketorolac) being specifically recommended for reducing pain intensity in cancer patients [16]. These factors collectively likely explain the surprising reduction in GABA usage leading into 2020. Regardless, caution is warranted in using NSAIDs for older cancer survivors, given that the age-related physiological changes amplify gastrointestinal, CNS, and renal toxicities of NSAIDs in older adults.

Our findings of opioid-naïve patients experiencing a sharper decline in opioid use and a greater increase in GABA prescriptions are consistent with past research, suggesting a shift toward non-opioid (and presumably safer) alternatives for initial pain management. The increase in GABA prescribing for opioid-naïve patients is consistent with government and medical society guidelines discouraging the initiation of opioids in opioid-naïve individuals [21,32,40]. In contrast, opioid non-naïve patients, already on long-term opioid therapy, present with more complex and nuanced clinical needs, requiring multimodal strategies that include continued opioid use, albeit at reduced doses, along with GABAs, other CNS-active medications, and non-drug approaches.

Opioid-naïve patients demonstrated stable or increased SSRI use, while opioid non-naïve patients showed a decline in use, possibly due to concerns about polypharmacy-related drug–drug interactions (e.g., serotonergic syndrome risk for some opioids when used with SSRIs) [41,42,43]. It is also possible that clinicians may be prescribing SNRI more as an initial non-opioid analgesic and non-BZD anxiolytic drugs for opioid non-naïve patients with co-occurring pain and anxiety [32]. The decrease in BZD use among opioid non-naïve patients likely reflects the increased awareness of the overdose risks associated with opioid/BZD co-prescribing, as also highlighted in the CDC Clinical Practice Guidelines of 2016 [32]. The different findings in drug use patterns and trends by opioid naïvety vs. non-naïvety suggest the need for consideration of prior opioid use (naïvety vs. non-naïvety) in future studies and underscore the need to tailor treatment strategies for pain and anxiety based on opioid history. Because claims do not capture non-pharmacologic therapies, the observed reduction in BZD and opioids alongside the rising GABA/SNRI use may reflect pharmacological substitution rather than a greater uptake of guideline-emphasized non-pharmacologic therapies [16].

Variation by region in medication prescribing offers an opportunity to inform the development of safe-use policy to guide physicians and other prescribers of these powerful agents. It is important to consider that the observed variation in medication trends across different US regions offers opportunities to develop evidence-informed policy and practice guidelines for improving consistency and quality of care for older cancer survivors living with co-occurring pain and anxiety [44]. Especially in states with lower adoption rates for GABA and SNRI prescriptions, targeted education and policy adjustments can encourage prescribing practices to improve care consistency and reduce opioid/BZD co-prescribing. Notably, there is currently no federally mandated CMS tools to guide deprescribing in older adults, despite recommendations of routine medication review by the American Geriatrics Society as key to preventing inappropriate prescribing in older adults [45]. This is an important area for future research, to understand barriers and facilitators of incorporating routine medication review and deprescribing as part of optimal cancer survivorship care.

This study has several limitations. First, we did not include other non-SSRI and non-SNRI anxiolytic drugs (mirtazapine and buspirone) as alternatives to BZD. Second, we did not include the use of non-pharmacological approaches (physical therapy, psychotherapy, acupuncture, or injections) for managing pain and anxiety in older cancer survivors. Third, similar to other studies using administrative claims data, our analysis cannot confirm if medications were actually used and did not capture the medication used during hospitalizations. Claims data incompletely captures ongoing systemic therapy (e.g., hormonal or immunotherapy), which may influence symptom burden and prescribing, nor can it establish lifetime opioid naivety within opioid-naive subgroup. Fourth, we did not include patient-reported outcomes (e.g., pain severity or anxiety levels), which could offer critical context for understanding the comparative effectiveness of the prescribed treatments. Fifth, our findings may not be applicable to younger cancer survivor populations, or to individuals in HMOs, or to those not diagnosed within SEER regions. Sixth, using Medicare claims to identify depressive, anxiety, and drug use disorders has limitations, as these disorders are underreported, and treatment-seeking behavior may be influenced by stigma and historically high copays, leading to biased samples. Seventh, our survivorship cohort includes individuals living in advanced or progressive cancers, which have distinct trajectories of pain, anxiety, and treatment needs, which can affect prescribing practices. Thus, claims data may lack sensitivity and limit generalizability of findings for these disorders [46,47,48]. Lastly, regional differences in healthcare access, provider distribution, socioeconomic factors, and patient preferences that may affect prescribing patterns were not explored in this analysis, an important area for future research.

Future research should also include co-prescribing with CNS-active medications, non-pharmacological approaches, and patient-reported outcomes. Findings from such studies can guide patient-centered therapeutic decision-making for pain and anxiety management in older cancer survivors. Additionally, the impact on prescribing behaviors of new policies and public educational campaigns should be monitored and periodically evaluated for effectiveness and unintended consequences. This information will provide data to inform any needed changes to policy and practice guidelines, aimed at ensuring safe and effective medication prescribing for older cancer survivors, a rapidly rising segment of the older US population.

## Figures and Tables

**Table 1:** Descriptive characteristics of older cancer survivors within each calendar year (2013, 2016, 2018, 2020).

Variables	2013 (*n* = 281,168)	2016 (*n* = 397,369)	2018 (*n* = 381,601)	2020 (*n* = 359,097)
Age				
mean (std)	77.6 (7.3)	77.6 (7.3)	77.8 (7.3)	78.2 (7.4)
median (IQR)	77.0 (72.0, 83.0)	77.0 (72.0, 83.0)	77.0 (72.0, 83.0)	77.0 (72.0, 83.0)
Age, categorical				
66–74	111,333 (39.6)	157,955 (39.8)	145,367 (38.1)	131,360 (36.6)
75–84	116,008 (41.3)	163,628 (41.2)	162,445 (42.6)	154,057 (42.9)
85+	53,827 (19.1)	75,786 (19.1)	73,789 (19.3)	73,680 (20.5)
Years post-cancer diagnosis				
Mean (std)	7.6 (3.4)	9.0 (4.1)	10.3 (4.4)	12.0 (4.5)
Median (IQR)	7.0 (5.0, 10.0)	9.0 (6.0, 12.0)	10.0 (7.0, 14.0)	12.0 (8.0, 16.0)
Gender				
Male	149,342 (53.1)	215,658 (54.3)	204,782 (53.7)	189,403 (52.7)
Female	131,826 (46.9)	181,711 (45.7)	176,819 (46.3)	169,694 (47.3)
Race and Ethnicity				
Hispanic	21,894 (7.8)	25,314 (6.4)	24,720 (6.5)	22,755 (6.3)
NHB	20,059 (7.1)	28,178 (7.1)	25,320 (6.6)	22,054 (6.1)
NHW	225,164 (80.1)	324,992 (81.8)	311,409 (81.6)	294,233 (81.9)
NH Others	14,051 (5.0)	18,885 (4.8)	20,152 (5.3)	20,055 (5.6)
Cancer diagnosis				
Breast	89,279 (31.8)	129,550 (32.6)	129,879 (34.0)	128,550 (35.8)
Colorectal	49,513 (17.6)	62,416 (15.7)	57,635 (15.1)	51,989 (14.5)
Lung	18,881 (6.7)	24,674 (6.2)	21,860 (5.7)	18,406 (5.1)
Prostate	123,495 (43.9)	180,729 (45.5)	172,227 (45.1)	160,152 (44.6)
Diagnosis cohort				
2000–2003	91,889 (32.7)	99,202 (25.0)	84,464 (22.1)	74,091 (20.6)
2004–2007	101,418 (36.1)	112,943 (28.4)	98,691 (25.9)	88,955 (24.8)
2008–2011	83,641 (29.8)	118,612 (29.9)	108,257 (28.4)	101,296 (28.2)
2012–2015	4220 (1.5)	66,612 (16.8)	90,189 (23.6)	94,755 (26.4)
Census region *				
Midwest	22,132 (7.9)	31,161 (7.8)	28,289 (7.4)	25,737 (7.2)
Northeast	94,608 (33.6)	140,235 (35.3)	132,379 (34.7)	123,681 (34.4)
South	85,964 (30.6)	117,379 (29.5)	110,746 (29.0)	103,225 (28.7)
West	78,365 (27.9)	108,443 (27.3)	110,062 (28.8)	106,245 (29.6)
Urban-rural status				
Metro	239,065 (85.0)	342,713 (86.3)	331,230 (86.8)	313,068 (87.2)
Non-Metro	42,041 (15.0)	54,572 (13.7)	50,298 (13.2)	45,969 (12.8)
Unknown	62 (0.0)	84 (0.0)	73 (0.0)	60 (0.0)
Original reason for enrollment				
Disability	27,061 (9.6)	36,041 (9.1)	34,504 (9.0)	31,900 (8.9)
Not disability related	254,107 (90.4)	361,328 (90.9)	347,097 (91.0)	327,197 (91.1)
Dual eligibility				
Yes	64,312 (22.9)	63,790 (16.1)	59,736 (15.7)	52,866 (14.7)
No	216,856 (77.1)	333,579 (83.9)	321,865 (84.4)	306,231 (85.3)
Charlson comorbidity => 1	192,886 (68.6)	269,970 (67.9)	258,751 (67.8)	245,176 (68.3)
Depressive disorder	32,962 (12.4)	53,326 (12.2)	53,500 (14.0)	55,797 (15.5)
Anxiety disorder	27,752 (9.9)	49,190 (12.4)	54,607 (14.3)	57,067 (15.9)
Alcohol use disorder	3944 (1.4)	7664 (1.9)	5829 (1.5)	5887 (1.6)
Drug use disorder	2333 (0.8)	5652 (1.4)	7372 (1.9)	8358 (2.3)
Opioid naïve **	189,083 (67.3)	277,532 (69.8)	278,559 (73.0)	277,098 (77.2)

* Exclude other regions due to small sample size. ** Opioid naïve status denotes absence of recorded prescriptions during the 12-month look-back. This does not imply lifetime opioid naivete.

**Table 2:** Adjusted Odds Ratios (aOR) and 95% Confidence Intervals (CI) for the receipt of GABA, Benzodiazepine, SSRI, SNRI, and opioid medications among older cancer survivors by various demographic, clinical, and temporal factors).

		GABA aOR (95% CI)	BENZO aOR (95% CI)	SSRI aOR (95% CI)	SNRI aOR (95% CI)	OPIOID aOR (95% CI)
Years post-cancer diagnosis		0.99 (0.99, 1.00)	1.01 (1.00, 1.01)	1.02 (1.01, 1.02)	1.01 (0.99, 1.01)	1.01 (1.00, 1.01)
calendar year	2014	1.09 (1.08, 1.11)	0.98 (0.97, 0.99)	0.99 (0.98, 1.00)	1.05 (1.03, 1.07)	0.98 (0.96, 0.99)
	2015	1.15 (1.13, 1.17)	0.98 (0.97, 1.00)	0.98 (0.97, 1.00)	1.06 (1.03, 1.09)	0.90 (0.88, 0.92)
	2016	1.23 (1.21, 1.26)	0.93 (0.91, 0.94)	0.97 (0.95, 0.99)	1.09 (1.05, 1.12)	0.84 (0.83, 0.86)
	2017	1.31 (1.28, 1.35)	0.87 (0.84, 0.89)	0.98 (0.96, 1.00)	1.14 (1.09, 1.19)	0.80 (0.78, 0.82)
	2018	1.37 (1.33, 1.42)	0.81 (0.79, 0.84)	0.98 (0.95, 1.01)	1.19 (1.14, 1.25)	0.72 (0.70, 0.74)
	2019	1.40 (1.35, 1.45)	0.75 (0.72, 0.78)	0.99 (0.96, 1.02)	1.25 (1.18, 1.32)	0.62 (0.60, 0.64)
	2020	1.34 (1.28, 1.40)	0.71 (0.68, 0.74)	0.99 (0.95, 1.02)	1.25 (1.17, 1.34)	0.59 (0.57, 0.62)
age	75–84	1.08 (1.07, 1.09)	1.01 (0.99, 1.01)	1.02 (1.01, 1.03)	0.86 (0.84, 0.87)	1.06 (1.05, 1.08)
	85+	0.99 (0.97, 1.01)	0.97 (0.95, 0.98)	1.12 (1.11, 1.14)	0.69 (0.67, 0.71)	1.14 (1.13, 1.16)
Gender	Female	1.18 (1.15, 1.21)	1.59 (1.55, 1.64)	1.73 (1.68, 1.77)	1.86 (1.77, 1.95)	1.25 (1.22, 1.28)
Race and Ethnicity	Hispanics	1.01 (0.98, 1.03)	0.75 (0.73, 0.77)	0.71 (0.69, 0.72)	0.62 (0.59, 0.65)	0.84 (0.82, 0.86)
	NH Unknowns/Others	0.85 (0.83, 0.88)	0.50 (0.48, 0.52)	0.41 (0.40, 0.43)	0.42 (0.39, 0.45)	0.54 (0.52, 0.56)
	NHB	1.03 (1.00, 1.05)	0.44 (0.43, 0.46)	0.40 (0.39, 0.41)	0.45 (0.43, 0.47)	1.02 (1.00, 1.04)
Cancer diagnosis	Breast	0.84 (0.82, 0.86)	0.73 (0.71, 0,75)	0.87 (0.85, 0.89)	1.11 (1.07, 1.15)	0.76 (0.74, 0.78)
	Colorectal	0.81 (0.78, 0.83)	0.66 (0.64, 0.68)	0.78 (0.76, 0.80)	0.79 (0.76, 0.83)	0.77 (0.75, 0.79)
	Prostate	0.71 (0.69, 0.73)	0.68 (0.66, 0.71)	0.81 (0.78, 0.83)	0.89 (0.85, 0.94)	0.70 (0.69, 0.72)
Diagnosis cohort	2004–2007	1.01 (0.98, 1.04)	1.02 (0.99, 1.05)	1.02 (1.00, 1.05)	0.98 (0.94, 1.03)	0.99 (0.97, 1.02)
	2008–2011	1.01 (0.95, 1.05)	1.03 (0.98, 1.09)	1.03 (0.99, 1.08)	0.99 (0.92, 1.06)	0.99 (0.95, 1.04)
	2012–2015	0.99 (0.92, 1.06)	1.05 (0.98, 1.13)	1.06 (0.99, 1.13)	1.02 (0.92, 1.14)	0.96 (0.90, 1.03)
Census region	NorthEast	0.83 (0.81, 0.86)	1.04 (1.01, 1.06)	0.95 (0.93, 0.97)	0.86 (0.83, 0.90)	0.65 (0.64, 0.67)
	South	1.24 (1.21, 1.27)	1.15 (1.12, 1.17)	1.11 (1.09, 1.14)	1.22 (1.18, 1.27)	0.98 (0.96, 1.00)
	West	1.07 (1.05, 1.10)	0.98 (0.96, 1.01)	0.92 (0.90, 0.94)	1.03 (0.99, 1.08)	0.94 (0.91, 0.96)
Urban-rural status	Non-Metro	1.09 (1.07, 1.11)	0.91 (0.89, 0.93)	1.08 (1.06, 1.10)	0.91 (0.88, 0.94)	1.17 (1.15, 1.19)
	Unknown	0.92 (0.59, 1.44)	1.28 (0.84, 1.96)	0.86 (0.56, 1.32)	1.39 (0.79, 2.46)	1.29 (0.85, 1.94)
Original reason for entitlement	Disability	1.72 (1.69, 1.75)	1.71 (1.68, 1.75)	1.44 (1.42, 1.47)	1.76 (1.71, 1.81)	1.95 (1.92, 1.99)
Dual eligibility	Yes	1.53 (1.51, 1.55)	1.31 (1.29, 1.33)	1.48 (1.46, 1.50)	1.22 (1.19, 1.24)	1.70 (1.68, 1.73)
Charlson comorbidity ≥ 1		1.33 (1.32, 1.34)	1.09 (1.08, 1.10)	1.12 (1.11, 1.13)	1.11 (1.10, 1.13)	1.38 (1.36, 1.40)
Depressive disorder		1.19 (1.18, 1.20)	1.24 (1.22, 1.25)	1.51 (1.50, 1.53)	1.59 (1.56, 1.61)	1.27 (1.26, 1.29)
Anxiety disorder		1.10 (1.09, 1.11)	1.56 (1.54, 1.57)	1.22 (1.21, 1.23)	1.18 (1.16, 1.20)	1.26 (1.25, 1.28)
Alcohol use disorder		1.06 (1.03, 1.09)	0.98 (0.95, 1.00)	1.04 (1.02, 1.06)	1.05 (1.01, 1.09)	0.95 (0.92, 0.98)
Drug use disorder		1.26 (1.23, 1.29)	1.13 (1.10, 1.15)	1.01 (0.99, 1.03)	1.17 (1.13, 1.21)	2.79 (2.73, 2.86)
Opioid naive		0.70 (0.69, 0.70)	0.85 (0.84, 0.85)	0.94 (0.94, 0.95)	0.80 (0.79, 0.81)	0.14 (0.14, 0.14)

**Table 3:** Adjusted Odds Ratios (aOR) and 95% Confidence Intervals (CI) for the Receipt of GABA, Benzodiazepine, SSRI, SNRI, and opioid medications among older cancer survivors by opioid naivety. *p*-value interaction between opioid naivety and calendar year is <0.0001.

		GABA aOR (95% CI)	BENZO aOR (95% CI)	SSRI aOR (95% CI)	SNRI aOR (95% CI)	OPIOID aOR (95% CI)
Opioid Naive						
Calendar Year	2014	1.12 (1.10, 1.14)	0.99 (0.98, 1.01)	0.99 (0.98, 1.01)	1.03 (1.01, 1.06)	0.97 (0.94, 1.01)
	2015	1.27 (1.24, 1.30)	1.02 (1.01, 1.04)	1.01 (0.98, 1.02)	1.07 (1.04, 1.11)	0.95 (0.92, 0.98)
	2016	1.38 (1.34, 1.42)	0.97 (0.94, 0.99)	0.99 (0.97, 1.01)	1.09 (1.05, 1.14)	0.84 (0.81, 0.87)
	2017	1.48 (1.43, 1.53)	0.91 (0.88, 0.94)	1.01 (0.98, 1.03)	1.16 (1.10, 1.23)	0.77 (0.74, 0.80)
	2018	1.56 (1.50, 1.63)	0.86 (0.83, 0.90)	1.01 (0.98, 1.04)	1.22 (1.15, 1.30)	0.65 (0.62, 0.68)
	2019	1.62 (1.54, 1.69)	0.80 (0.77, 0.84)	1.02 (0.99, 1.06)	1.29 (1.20, 1.38)	0.51 (0.48, 0.53)
	2020	1.55 (1.47, 1.63)	0.76 (0.72, 0.80)	1.02 (0.97, 1.06)	1.27 (1.17, 1.38)	0.47 (0.45, 0.50)
Non-Opioid Naïve *						
Calendar Year	2014	1.06 (1.04, 1.09)	0.94 (0.92, 0.96)	0.94 (0.92, 0.96)	1.05 (1.02, 1.09)	1.02 (1.00, 1.03)
	2015	1.08 (1.05, 1.11)	0.91 (0.89, 0.94)	0.91 (0.88, 0.93)	1.06 (1.02, 1.10)	0.98 (0.96, 1.01)
	2016	1.14 (1.11, 1.18)	0.85 (0.83, 0.88)	0.88 (0.86, 0.91)	1.10 (1.05, 1.16)	0.95 (0.93, 0.98)
	2017	1.23 (1.18, 1.27)	0.79 (0.76, 0.82)	0.88 (0.85, 0.91)	1.16 (1.09, 1.23)	0.92 (0.89, 0.95)
	2018	1.30 (1.24, 1.35)	0.74 (0.70, 0.77)	0.87 (0.83, 0.90)	1.23 (1.15, 1.31)	0.85 (0.82, 0.88)
	2019	1.32 (1.25, 1.38)	0.66 (0.63, 0.70)	0.85 (0.82, 0.90)	1.28 (1.19, 1.38)	0.76 (0.73, 0.79)
	2020	1.21 (1.14, 1.28)	0.61 (0.57, 0.65)	0.84 (0.80, 0.89)	1.30 (1.19, 1.41)	0.72 (0.69, 0.76)

* For the opioid outcome, the working correlation structure was specified as independent due to the non-convergence of the AR1 structure.

## Data Availability

The datasets used to conduct this study are available upon approval of a research protocol from the National Cancer Institute. Instructions for obtaining these data are available at https://healthcaredelivery.cancer.gov/seermedicare/obtain/ (accessed on 24 September 2024).

## Supplementary Materials

The following supporting information can be downloaded at: https://www.mdpi.com/article/10.3390/curroncol32100576/s1, Table S1: Adjusted Odds Ratios (aOR) and 95% Confidence Intervals (CI) for the Receipt of GABA, Benzodiazepine, SSRI, SNRI, and Opioid Medications Among Older Cancer Survivors by regions. *p*-value interaction between region and calendar year is <0.0001.

## Abbreviations

The following abbreviations are used in this manuscript:

aOR	Adjusted odds ratio
AR1	Autoregressive
BZD	Benzodiazepine
CI	Confidence Interval
CDC	Centers for Disease Control and Prevention
CMS	Centers for Medicare and Medicaid Services
CNS	Central nervous system
GABA	Gabapentinoids
GEE	Generalized estimating equations
HMO	Health Maintenance Organization
ICD	International Classification of Disease
NHB	Non-Hispanic Black
NHW	Non-Hispanic White
NSAID	Non-steroidal anti-inflammatory drugs
RECORD	Reporting of studies Conducted using Observational Routinely collected Data
SD	standard deviation
SEER	Surveillance, Epidemiology, and End Results
SNRI	Serotonin-Norepinephrine Reuptake Inhibitor
SSRI	Selective Serotonin Reuptake Inhibitor
