# Supplementary material for: Regional and Temporal Variation in Receipt of Gabapentinoid and SSRI/SNRI Therapy Among Older Cancer Survivors in the United States
Source: Curr Oncol. 2025 Oct 17;32(10):576. doi: 10.3390/curroncol32100576 (PMC12563467; doi:10.3390/curroncol32100576)
Supplement: Supplementary file 1 [file curroncol-32-00576-s001.zip › curroncol-3874476-supplementary.pdf]

Supplementary Table S1. Adjusted Odds Ratios (aOR) and 95% Confidence Intervals (CI) for the Receipt of GABA, Benzodiazepine, SSRI, SNRI, and Opioid Medications Among Older Cancer Survivors by regions. P-value interaction between region and calendar year is <0.0001.

		GABA aOR(95% CI)	BENZO aOR(95% CI)	SSRI aOR(95% CI)	SNRI aOR(95% CI)	OPIOID aOR(95% CI)
**West**						
calendar year	2014	1.08 (1.05, 1.11)	1.03 (1.01, 1.05)	0.98 (0.96, 1.01)	1.05 (1.01, 1.09)	0.99 (0.96, 1.02)
	2015	1.13 (1.09, 1.17)	1.02 (0.99, 1.05)	0.97 (0.95, 1.00)	1.06 (1.01, 1.12)	0.92 (0.89, 0.95)
	2016	1.20 (1.15, 1.25)	0.96 (0.92, 1.00)	0.96 (0.92, 1.00)	1.09 (1.03, 1.16)	0.85 (0.82, 0.89)
	2017	1.28 (1.22, 1.35)	0.89 (0.85, 0.94)	0.96 (0.92, 1.01)	1.14 (1.06, 1.24)	0.82 (0.79, 0.86)
	2018	1.35 (1.27, 1.43)	0.83 (0.78, 0.89)	0.96 (0.90, 1.01)	1.22 (1.11, 1.34)	0.75 (0.71, 0.79)
	2019	1.38 (1.29, 1.48)	0.75 (0.70, 0.81)	0.97 (0.91, 1.03)	1.26 (1.14, 1.41)	0.62 (0.58, 0.66)
	2020	1.33 (1.23, 1.44)	0.70 (0.65, 0.76)	0.95 (0.88, 1.03)	1.29 (1.14, 1.46)	0.60 (0.56, 0.65)
**South**						
calendar year	2014	1.10 (1.08, 1.13)	0.98 (0.96, 1.01)	0.99 (0.98, 1.02)	1.06 (1.03, 1.10)	0.99 (0.98, 1.02)
	2015	1.17 (1.14, 1.21)	0.97 (0.94, 0.99)	1.01 (0.98, 1.03)	1.07 (1.03, 1.12)	0.90 (0.88, 0.93)
	2016	1.25 (1.20, 1.29)	0.90 (0.87, 0.94)	0.99 (0.96, 1.03)	1.11 (1.05, 1.17)	0.87 (0.84, 0.90)
	2017	1.31 (1.25, 1.37)	0.86 (0.82, 0.90)	0.99 (0.96, 1.04)	1.16 (1.09, 1.24)	0.82 (0.78, 0.85)
	2018	1.35 (1.28, 1.42)	0.80 (0.76, 0.85)	0.99 (0.94, 1.04)	1.21 (1.12, 1.31)	0.75 (0.72, 0.79)
	2019	1.39 (1.31, 1.48)	0.73 (0.69, 0.78)	1.01 (0.94, 1.06)	1.27 (1.16, 1.40)	0.64 (0.60, 0.68)
	2020	1.33 (1.24, 1.42)	0.69 (0.64, 0.74)	1.01 (0.94, 1.08)	1.28 (1.15, 1.43)	0.62 (0.58, 0.66)
**Midwest**						
calendar year	2014	1.10 (1.04, 1.15)	0.99 (0.95, 1.05)	1.01 (0.98, 1.05)	1.06 (0.98, 1.14)	0.99 (0.94, 1.04)
	2015	1.14 (1.07, 1.22)	1.11 (1.04, 1.18)	1.01 (0.95, 1.06)	1.08 (0.98, 1.19)	0.89 (0.84, 0.94)
	2016	1.22 (1.12, 1.32)	1.04 (0.96, 1.12)	0.99 (0.93, 1.06)	1.16 (1.02, 1.31)	0.85 (0.79, 0.91)
	2017	1.28 (1.16, 1.42)	0.95 (0.86, 1.05)	0.99 (0.91, 1.12)	1.26 (1.08, 1.48)	0.81 (0.75, 0.88)
	2018	1.37 (1.22, 1.54)	0.86 (0.76, 0.96)	1.01 (0.91, 1.12)	1.34 (1.11, 1.62)	0.68 (0.62, 0.76)
	2019	1.32 (1.15, 1.51)	0.77 (0.67, 0.88)	1.01 (0.89, 1.14)	1.44 (1.16, 1.79)	0.59 (0.53, 0.66)
	2020	1.22 (1.04, 1.43)	0.74 (0.63, 0.87)	1.01 (0.88, 1.17)	1.44 (1.12, 1.86)	0.59 (0.52, 0.67)
**Northeast**						
calendar year	2014	1.09 (1.06, 1.12)	0.95 (0.93, 0.97)	0.98 (0.96, 0.99)	1.02 (0.98, 1.05)	0.93 (0.91, 0.96)
	2015	1.16 (1.12, 1.19)	0.94 (0.92, 0.97)	0.97 (0.94, 0.99)	1.03 (0.98, 1.08)	0.88 (0.85, 0.91)

2016	1.26<br>(1.20, 1.31)	0.90<br>(0.86, 0.93)	0.96<br>(0.93, 0.99)	1.03<br>(0.97, 1.10)	0.80<br>(0.77, 0.84)
2017	1.36<br>(1.29, 1.43)	0.84<br>(0.80, 0.88)	0.97<br>(0.94, 1.01)	1.09<br>(1.02, 1.18)	0.75<br>(0.71, 0.78)
2018	1.43<br>(1.35, 1.52)	0.80<br>(0.76, 0.84)	0.98<br>(0.93, 1.03)	1.12<br>(1.03, 1.23)	0.67<br>(0.63, 0.71)
2019	1.46<br>(1.36, 1.56)	0.75<br>(0.71, 0.80)	0.99<br>(0.94, 1.05)	1.17<br>(1.05, 1.30)	0.59<br>(0.56, 0.64)
2020	1.39<br>(1.29, 1.50)	0.72<br>(0.67, 0.77)	0.99<br>(0.93, 1.06)	1.15<br>(1.02, 1.30)	0.56<br>(0.52, 0.60)
